# Supplementary material for: Multi‐dataset identification of innovative feature genes and molecular mechanisms in keratoconus
Source: J Cell Mol Med. 2024 Sep 19;28(18):e70079. doi: 10.1111/jcmm.70079 (PMC11412914; doi:10.1111/jcmm.70079)
Supplement: Supplementary file 2 — Material S2. [file JCMM-28-e70079-s002.zip › 7.GSEA analysis/1.ARL11/KEGG_CARDIAC_MUSCLE_CONTRACTION.html]

Details for gene set KEGG\_CARDIAC\_MUSCLE\_CONTRACTION[GSEA]

|  || Dataset | ARL11.group.cls#h\_versus\_l |
| Phenotype | group.cls#h\_versus\_l |
| Upregulated in class | h |
| GeneSet | KEGG\_CARDIAC\_MUSCLE\_CONTRACTION |
| Enrichment Score (ES) | 0.25727364 |
| Normalized Enrichment Score (NES) | 0.9609439 |
| Nominal p-value | 0.5562249 |
| FDR q-value | 1.0 |
| FWER p-Value | 1.0 |
Table: GSEA Results Summary

  

Fig 1: Enrichment plot: KEGG\_CARDIAC\_MUSCLE\_CONTRACTION      
 Profile of the Running ES Score & Positions of GeneSet Members on the Rank Ordered List

  

| SYMBOL | TITLE | RANK IN GENE LIST | RANK METRIC SCORE | RUNNING ES | CORE ENRICHMENT || 1 | TNNI3 | na | 44 | 0.763 | 0.0946 | Yes |
| 2 | TNNT2 | na | 262 | 0.553 | 0.1579 | Yes |
| 3 | CACNB1 | na | 1069 | 0.381 | 0.1825 | Yes |
| 4 | CACNG8 | na | 1424 | 0.344 | 0.2156 | Yes |
| 5 | CACNA1F | na | 1554 | 0.333 | 0.2536 | Yes |
| 6 | ATP1B4 | na | 2566 | 0.261 | 0.2573 | Yes |
| 7 | ACTC1 | na | 5327 | 0.156 | 0.1973 | No |
| 8 | TPM2 | na | 5376 | 0.155 | 0.2154 | No |
| 9 | CACNA2D4 | na | 6234 | 0.131 | 0.2071 | No |
| 10 | ATP1B1 | na | 6792 | 0.117 | 0.2057 | No |
| 11 | CACNG5 | na | 8337 | 0.086 | 0.1719 | No |
| 12 | MYH6 | na | 8587 | 0.081 | 0.1750 | No |
| 13 | CACNA2D3 | na | 8962 | 0.075 | 0.1737 | No |
| 14 | ATP2A2 | na | 9649 | 0.065 | 0.1620 | No |
| 15 | TPM1 | na | 10619 | 0.052 | 0.1406 | No |
| 16 | TPM4 | na | 11422 | 0.042 | 0.1228 | No |
| 17 | COX7A2L | na | 12931 | 0.028 | 0.0828 | No |
| 18 | COX6B2 | na | 13815 | 0.020 | 0.0599 | No |
| 19 | ATP1A1 | na | 13911 | 0.020 | 0.0596 | No |
| 20 | UQCRQ | na | 15016 | 0.011 | 0.0291 | No |
| 21 | COX6B1 | na | 15603 | 0.007 | 0.0131 | No |
| 22 | SLC9A6 | na | 15787 | 0.005 | 0.0084 | No |
| 23 | COX8A | na | 16384 | 0.001 | -0.0086 | No |
| 24 | UQCRC1 | na | 16414 | 0.001 | -0.0094 | No |
| 25 | COX7B | na | 16679 | -0.001 | -0.0168 | No |
| 26 | MYH7 | na | 16998 | -0.003 | -0.0256 | No |
| 27 | COX5B | na | 17270 | -0.005 | -0.0328 | No |
| 28 | UQCRC2 | na | 17359 | -0.006 | -0.0346 | No |
| 29 | UQCR11 | na | 17430 | -0.006 | -0.0358 | No |
| 30 | COX7C | na | 17486 | -0.007 | -0.0366 | No |
| 31 | COX7B2 | na | 18020 | -0.010 | -0.0506 | No |
| 32 | COX7A2 | na | 18098 | -0.011 | -0.0515 | No |
| 33 | TPM3 | na | 18375 | -0.013 | -0.0578 | No |
| 34 | CACNG3 | na | 18568 | -0.014 | -0.0616 | No |
| 35 | COX6C | na | 19016 | -0.017 | -0.0724 | No |
| 36 | COX6A1 | na | 19083 | -0.017 | -0.0721 | No |
| 37 | CACNB4 | na | 19492 | -0.020 | -0.0813 | No |
| 38 | UQCRH | na | 19528 | -0.021 | -0.0797 | No |
| 39 | CACNA1D | na | 19640 | -0.021 | -0.0802 | No |
| 40 | ATP1B3 | na | 19840 | -0.023 | -0.0831 | No |
| 41 | COX4I1 | na | 20260 | -0.026 | -0.0920 | No |
| 42 | ATP1A3 | na | 20328 | -0.026 | -0.0906 | No |
| 43 | UQCRFS1 | na | 20848 | -0.030 | -0.1018 | No |
| 44 | UQCR10 | na | 21044 | -0.032 | -0.1034 | No |
| 45 | COX5A | na | 21246 | -0.033 | -0.1051 | No |
| 46 | CACNB2 | na | 22149 | -0.041 | -0.1259 | No |
| 47 | CACNG1 | na | 22207 | -0.042 | -0.1223 | No |
| 48 | UQCRB | na | 22209 | -0.042 | -0.1170 | No |
| 49 | CYC1 | na | 22402 | -0.044 | -0.1170 | No |
| 50 | FXYD2 | na | 22512 | -0.045 | -0.1145 | No |
| 51 | ATP1A4 | na | 22955 | -0.049 | -0.1210 | No |
| 52 | ATP1B2 | na | 23314 | -0.054 | -0.1246 | No |
| 53 | CACNG2 | na | 23379 | -0.054 | -0.1196 | No |
| 54 | TNNC1 | na | 24398 | -0.068 | -0.1405 | No |
| 55 | CACNA1C | na | 25293 | -0.080 | -0.1563 | No |
| 56 | MYL3 | na | 26146 | -0.093 | -0.1692 | No |
| 57 | CACNA2D2 | na | 26358 | -0.096 | -0.1632 | No |
| 58 | COX4I2 | na | 26565 | -0.100 | -0.1566 | No |
| 59 | CACNB3 | na | 26714 | -0.103 | -0.1480 | No |
| 60 | CACNG7 | na | 27851 | -0.127 | -0.1648 | No |
| 61 | COX7A1 | na | 28636 | -0.147 | -0.1689 | No |
| 62 | SLC8A1 | na | 29232 | -0.164 | -0.1655 | No |
| 63 | ATP1A2 | na | 29374 | -0.168 | -0.1485 | No |
| 64 | CACNG4 | na | 29566 | -0.174 | -0.1321 | No |
| 65 | CACNA2D1 | na | 29712 | -0.179 | -0.1138 | No |
| 66 | SLC9A1 | na | 30058 | -0.190 | -0.0999 | No |
| 67 | COX6A2 | na | 30087 | -0.190 | -0.0768 | No |
| 68 | MYL2 | na | 30743 | -0.212 | -0.0690 | No |
| 69 | CACNG6 | na | 31816 | -0.253 | -0.0681 | No |
| 70 | RYR2 | na | 33835 | -0.395 | -0.0768 | No |
| 71 | CACNA1S | na | 33887 | -0.403 | -0.0276 | No |
| 72 | COX8C | na | 33951 | -0.412 | 0.0224 | No |
Table: GSEA details [plain text format]

  

Fig 2: KEGG\_CARDIAC\_MUSCLE\_CONTRACTION      
 Blue-Pink O' Gram in the Space of the Analyzed GeneSet

  

Fig 3: KEGG\_CARDIAC\_MUSCLE\_CONTRACTION: Random ES distribution      
 Gene set null distribution of ES for **KEGG\_CARDIAC\_MUSCLE\_CONTRACTION**
